# Uterine Diseases

**Published:** 1868-10-01

**Authors:** 


### *PHILADELPHIA CORRESPONDENCE.*

### UTERINE DISEASES.

PHILADELPHIA, *September 24th*, 1868.

"THE diseases in question, (uterine,) are those to which females are *most commonly exposed.* * * Inflammation of the neck of the uterus is an *exceedingly common disease.* * * In reality, inflammation is comparatively quite as frequent in the uterine system as in other similarly organized organs." Thus writes Bennet; and his assertions are amply vindicated by statistics of the present day. Notice the clinics at our various colleges, and we shall find, if we examine with impartiality, that a very large proportion of females applying for treatment are affected with uterine disease of some character and degree. The prevailing affections — at least so far as our observations, clinical and otherwise, goes — are inflammation and ulcerations. With these, there is, as a necessary accompaniment, more or less hypertrophy. The prevalence of uterine affections in our cities is unprecedented. Indeed some have gone so far as to assert that "three-fifths of our females" are so diseased. If we make an examination, we shall perhaps find slight inflammation — but a faint blush tinging the uterus — or it may be congestion deep and full has set in, or still further ulceration may be decided. With whatever of these degrees or characters of disease the uterus may be attacked, the general train of symptoms is identical. No local symptom will indicate the disease, but that dull and dragging pain in the back and loins, that pain between the shoulders, the indigestion and constipation of the bowels, and the peculiar cephalic symptoms, themselves so diagnostic of uterine affection, all lead us to a positive conclusion that there is disease of the uterus or its appendages. Why *is* this disease so markedly prevalent? What are the causes of uterine disease? The anatomical predisposing cause of all uterine inflammatory diseases, is especially the great vitality and vascularity of the neck, as well as the highly developed mucous membrane lining that cervix. This fact remembered, the *incidental* causes are to a degree manifest. First, should be ranked parturition. Marriage in the United States takes place early in life. The wife is a mother ere she has fairly reached womanhood. The uterus is lashed into excitement early, and metritis results. Again, "*fashionable woman,*" ("*stylish*") during her menstrual period, adorns herself for the ball-room, where she revels in dance, heated and fatigued, to gain for herself dysmenorrhœa, consequent probably upon inflammation of the cervix. Further, this same *personification of the elite* must lace her waist to a span, that *her* form (?) may be remarked upon. From this anteversion or retroversion is the result. Still *another* phase in this *fashionable woman's* life. In order that she may be present at *all* the parties, balls and operas, she mustn't have a *baby* to tie her to her home. "Oh, my, it's not fashionable to have a child!" she says, and if perchance, pregnancy occurs, off she hastes to some murderess or beast, and from her she *buys* an abortion. Inflammation, ulceration, and perhaps even death ends her *fashionable career!* This is no overdrawn picture. It is the *real truth*, but God forbid that *such* style, *such* fashion should prevail among the intelligent women of this country! And what is the natural result of this condition? What evil comes out of it? In my last letter I stated that it "prevented, to an alarming extent, the furtherance of the race." It is true that abortions are a frequent *cause* of uterine affections, but it is as true that they are the *results* of this class of diseases. Sterility is by no means an uncommon complaint among women. It is an abnormal condition of nature. Why is it so common? It is simply because uterine diseases are so prevalent, and no cure is instituted for it. Country practitioners, as a class, give medicines for the symptoms indicative of uterine affections. In additions to the various diseases of the uterus *preventing* pregnancy, they are the constant cause of miscarriage and abortions, thus by two ways fulfilling the same end, namely: arresting the promotion of the race. A case occurred in our clinic a short time ago, which illustrates this point. A woman, thirty-two years old, had had five miscarriages. Rest and inclined posture had no effect in thwarting the abortion. A vaginal examination revealed an ulcer about the os uteri. A few applications of *Argent. nit.* cured the disease, and the woman is now a mother. Another case under my own observation, in which the patient, aged thirty-seven, had had *thirteen* miscarriages. Four applications of *Argent. nit.* healed an abraded surface of cervix, and the woman is now a mother of two living children, and is five months *enciente*. If such and kindred treatment can cure these cases, is it not well to devote *special* care and time to it? I shall illustrate the subject more fully in my next letter, by giving some interesting cases. Yours, E. R. H.

